# Neighborhood walkability and sedentary behaviors among US adults: Erratum

**DOI:** 10.1097/EE9.0000000000000466

**Published:** 2026-02-16

**Authors:** Yangyang Deng, Mohammad Moniruzzaman, Breanna Rogers, Jinani Jayasekera, Lu Hu, Tomoki Nakaya, Eran Ben-Joseph, David Berrigan, Charles E Matthews, Kosuke Tamura

In the article beginning on page e453 in the January 2026 compendium of the journal, the authors identified two errors in Table 2.

First, for transport-related sedentary behavior, the upper limit of the confidence interval for the below average category was incorrectly reported as “−0.02” and should be “0.02.”

Second, for transport-related sedentary behavior, the upper limit of the confidence interval for the above average category was incorrectly reported as “−0.00” and should be “0.00.”

In addition, Reference 1 should have read as follows:

Sedentary Behaviour Research Network. Letter to the editor: standardized use of the terms “sedentary” and “sedentary behaviours”. *Appl Physiol Nutr Metab.* 2012;37:540–542.
